# Adult age differences in the modulation of peripersonal space after tool use in virtual reality

**DOI:** 10.1038/s41598-026-41116-y

**Published:** 2026-02-23

**Authors:** Dariusz O’Leary, Yichen Fan, Jens Krzywinski, Shu-Chen Li

**Affiliations:** 1 6G-life Research Hub, TU Dresden, Dresden, Germany; 2 Chair of Lifespan Developmental Neuroscience, Faculty of Psychology, TU Dresden, Dresden, Germany; 3 Centre for Tactile Internet with Human-in-the-Loop (CeTI), TU Dresden, Dresden, Germany; 4 Chair of Industrial Design Engineering, TU Dresden, Dresden, Germany

**Keywords:** Peripersonal space, virtual reality, tool use, aging, multisensory integration, Neuroscience, Psychology, Psychology

## Abstract

Tool use has been shown to modulate the multisensory representation of the area surrounding our body known as peripersonal space (PPS). Yet, it remains unclear whether such effects extend to tool use in virtual reality (VR), and whether age may influence this modulation of PPS in VR. We investigated these questions in younger adults (YAs; 19–29 years) and older adults (OAs; 65–84 years). Participants completed a tool-use task in VR while viewing a virtual avatar from a first-person perspective. Tool-use effects on PPS were assessed using a visuo-tactile task before and after tool use, and participants rated their sense of ownership over the avatar at both time points. Results in YAs suggest a location-specific modulation of PPS after tool use. In contrast, OAs did not show a location-specific modulation but exhibited results suggesting a general enhancement of PPS within the VR environment. Interestingly, while both groups showed increased avatar ownership following tool use, only OAs demonstrated an association between this increase in ownership and increased overall multisensory facilitation in VR. These findings suggest that PPS remains responsive to VR environments in older age, but that the mechanisms underlying PPS modulation after tool use in VR differ with age.

**Supplementary Information:**

The online version contains supplementary material available at 10.1038/s41598-026-41116-y.

## Introduction

To interact effectively with our surroundings, we need an accurate neurocognitive representation of our body and the space immediately surrounding it, commonly referred to as peripersonal space (PPS). The representation of PPS was first identified through single-cell recordings in non-human primates, which revealed bimodal neurons that respond to both tactile stimulation on the body and visual stimulation in the area around the body^1^. These bimodal neurons were shown to have stronger responses to visual stimuli presented near the body than to those farther away^2–4^. A similar distance-dependent pattern of PPS has been observed on a cortical and behavioral level in humans, with stronger multisensory visuo-tactile integration near the body that gradually weakens as the distance of the visual stimulus from the body increases^5–7^. This graded representation of PPS is thought to support the selective integration of multisensory stimuli in relation to their action relevance, prioritizing stimuli that are most pertinent for goal-directed actions and defensive responses^5^. PPS has thus been proposed to play a significant role in guiding interactions with the environment^5,8^.

An important characteristic of PPS is its malleability. PPS is not a static representation but rather can be modulated by factors such as body representation, social context, and body-object interactions (see^8^ for a review of the factors influencing the representation of PPS). Given the proposed role of PPS in action guidance^5,8^, numerous studies have investigated how actions themselves affect PPS (e.g.,^9–12^). One type of action widely used to exemplify the malleability of PPS is tool use. Initial evidence for this once more came from single-cell recordings in non-human primates, which showed that using a tool leads to the receptive fields of bimodal visuo-tactile neurons encoding PPS around the hand to expand and encompass an extended space where the tool was used^13^. This modulation of the spatial distribution of multisensory integration following tool use has since been replicated in humans in both behavioral and electrophysiological studies^14–17^. These studies observed increased multisensory integration between the hand and the spatial region where the tool was used, supporting the interpretation that tool use modulates hand-centered PPS.

More recently, there has been growing interest in examining whether the effects of tool use on PPS observed in physical settings also occur when the tool use takes place within a virtual reality (VR) environment. The representation of PPS has been shown to be present within VR environments and can be modulated by VR-specific manipulations^18–22^. For example, it has been shown that PPS is responsive to the characteristics of virtual objects and agents approaching in a VR environment^22^. Furthermore, introducing a visuo-proprioceptive discrepancy between the real and a virtual hand has been found to enlarge PPS^19^, while a disconnected virtual hand presented at a distance in the VR scene can lead to a transfer of PPS to that location^20^. However, the malleability of PPS remains underexplored in other VR contexts, such as those involving tool use.

If tool use in VR modulates PPS similarly to how it does in the physical world, it may indicate a partial transfer of virtually acquired skills to similar real-world tasks. This is especially relevant given the promise VR shows for training purposes across education, industry, and medical settings^23–25^. Moreover, such findings would highlight the potential of VR as a powerful experimental method for investigating tool-use effects on PPS under controlled yet ecologically valid conditions.

Despite this potential, research into whether tool use in VR affects PPS is still in its early stages. An initial study reported an extension of PPS after tool use in the physical world but not after comparable tool use in VR^26^. However, a more recent study by Petrizzo et al. (2024)^27^ comparing various types of tool use in VR found an extension of PPS when a virtual tool was used in movements from far to near virtual space. These findings suggest that it is important to consider the specific motor routine employed and that, for motor routines involving a transition between far to near space, VR-based tool use can affect PPS.

A gap in the existing literature on whether tool use in VR affects PPS is the absence of an avatar body in the virtual environment. Instead, participants experienced a disembodied first-person perspective, with no visible body representation^26,27^. Given that PPS refers to the space immediately surrounding the body, including a virtual body visible from a first-person perspective may enable a more accurate and ecologically valid assessment of PPS within VR. This is especially relevant in light of previous findings suggesting that both the sense of ownership over a virtual avatar and the general extent of avatar embodiment can influence PPS (e.g.,^18,21,28,29^). Moreover, including an avatar would allow the investigation of how using a tool in VR may affect the sense of avatar ownership, given previous results suggesting that visuo-motor synchrony between a participant’s real movements and those of an avatar enhances the sense of avatar ownership^30^. This, in turn, would provide an opportunity to explore whether changes in avatar ownership following tool use may be associated with changes in PPS, offering deeper insight into the mechanisms underlying the adaptation of PPS in VR.

One hurdle of including avatar bodies in VR studies is the technical challenge of animating them in synchrony with the participant’s movements. However, combining inverse kinematics^31^ with positional data from VR trackers provides a reliable method for animating the body of avatars to follow participants’ movements. This approach has been shown to create a strong sense of embodiment^32^ and may improve the ecological validity of PPS research in VR, by giving participants a realistic sense of owning a body within the virtual environment.

A further gap in the current literature is the lack of age-related comparisons. Research examining the effects of tool use on PPS in older adults has largely been limited to clinical populations, particularly individuals with neurological impairments following stroke^33–35^, whereas studies in healthy populations have predominantly investigated younger adults^14–17^. Existing age comparisons between healthy younger and older adults conducted in physical environments have shown that older adults do not show a change in their perceived distance to targets following tool use like younger adults do, implying age differences in the effects of tool use on the remapping of spatial representations^36–38^. To date, it is not clear how the effect of tool use on PPS may differ between younger and older adults in VR settings. If VR is to be designed as an age-inclusive technology, it is vital that potential age differences in multisensory perception and adaptation in VR be elucidated^39^. This is underscored by the growing use of VR for assessing and training both cognitive and motor functions in older adults (e.g.,^40–43^). Understanding whether and how tool use in VR modulates PPS in older adults could provide guidance as to how VR interventions aimed at this population need to be designed to improve their usability and effectiveness.

To investigate these questions, we conducted an experiment in VR with a sample of younger adults (YAs; 19–29 years) and older adults (OAs; 65–84 years). Specifically, and in light of the findings from Petrizzo et al. (2024)^27^, we designed a tool-use task in VR that involved a motor routine in which participants used a virtual tool to move objects from far to near virtual space. PPS was assessed before and after tool use with a modified visuo-tactile task used to capture the spatial pattern of multisensory facilitation (more details below)^44–46^. To give participants a sense of ownership over a virtual body, an avatar body co-located with the participant’s own body was visible throughout the experiment. During the tool-use task the avatar’s right arm was animated to follow participants’ right-arm movements using controller-based movement tracking. The sense of avatar ownership was assessed before and after tool use using the ownership subscale of the Embodiment Questionnaire^47^.

To measure changes to PPS following tool use in VR, a modified version of the visuo-tactile task^44–46^ was completed before and after tool use. This task required participants to make speeded responses to a tactile stimulus on the back of the right hand during the presence or absence of a concurrent task-irrelevant visual stimulus that loomed toward the participant’s viewpoint (and therefore also toward the avatar) from varying distances. In line with the distance-dependent nature of PPS^5–7^, the response time to the tactile stimulus is expected to vary with the distance of the visual stimulus, with multisensory facilitation (i.e., faster responses compared to unimodal tactile trials) anticipated to increase the closer the visual stimulus is to the participant’s (and in this case also avatar’s) body. In studies conducted in physical environments, this distance-dependent spatial distribution of multisensory facilitation was shown to be altered after compared to before tool use, with increased facilitation at distances farther away from the body at which the tool was used^15–17^. This stronger integration of multisensory stimuli at the location of tool use is generally interpreted as a tool-induced modulation of PPS.

Based on findings in physical environments suggesting that tool use does not lead to a remapping of spatial representations in older adults as in younger adults^36–38^, we expected to find a difference in the effect of VR tool use on the spatial distribution of multisensory facilitation between YAs and OAs. This would be indicated by an interaction between measurement time point (before vs. after tool use), the distance of the visual stimulus (six possible distances from the participant), and age (YAs vs. OAs). Furthermore, in YAs, we expected to find a tool-use effect on the spatial distribution of multisensory facilitation. This would be indicated by an interaction between time point and distance indicating increased multisensory facilitation following tool use at the location at which the tool use occurred. On the other hand, for OAs, we did not expect an effect of tool use on the spatial distribution of multisensory facilitation. This was again based on the finding that tool use does not lead to a remapping of spatial representations in older adults^36–38^ and would be indicated by the absence of an interaction between time point and distance in OAs. Finally, given the suggested link between the sense of avatar ownership and PPS^18,28,29^, we wanted to examine avatar ownership scores at both time points and how they may be associated with potential modulations of PPS through VR tool use.

## Methods

### Participants

Our sample size analysis was based on a three-way interaction between the factors time point, visual stimulus distance, and age, as this represented the highest-order effect of interest in our design and was a prerequisite for subsequent age-specific analyses of time point and distance effects. The required sample size for detecting this interaction was estimated using MorePower 6.0^48^. As no previous studies have reported effect sizes for this specific interaction, we based our calculation on a medium effect size of *η*^*2*^ = 0.06 and a power level of 0.80^49^, which yielded a recommended sample size of 22 participants per age group. To account for potential dropouts and to accommodate the greater inter- and intraindividual variability in reaction times typically observed in older adults^50,51^, we recruited a total of 30 YAs and 30 OAs.

From this original sample, three participants were excluded due to reported nausea, two due to technical issues during data collection, and one due to amblyopia in one eye. This resulted in a final effective sample of 28 YAs (18 female, 10 male, mean age = 23.46 years, age range = 19–29 years) and 26 OAs (13 female, 13 male, mean age = 70.62 years, age range = 65–84 years). All participants were right-handed as assessed by the Edinburgh Handedness Inventory^52^, and reported normal or corrected-to-normal vision, no abnormalities in tactile perception, and no history of neurological or current psychiatric disorders. Participants received 15€ or course credit (if preferred by student participants) for participation in the study.

The study was approved by the ethics committee of TU Dresden (approval number: SR-EK-5012021) and all methods were performed in accordance with the relevant guidelines and regulations. Furthermore, all participants provided written informed consent prior to participation.

### Apparatus

The virtual environment and visual stimuli were developed using Unity 3D (version 2020.3.33f1; Unity Technologies, United States) and presented through a base HTC VIVE VR headset (Valve Corporation, United States). The experimental flow and trial structure were managed using the Unity Experiment Framework^53^.

Tactile stimuli were delivered via a vibrotactile motor (Vibrating Mini Motor Disc #1201, Adafruit, United States) of 10 mm diameter, vibrating at a frequency of 100 Hz, and for a duration of 100 ms. An ESP32-PICO-D4 microcontroller (Espressif Systems, China) was used to control the delivery of the tactile stimuli. The same microcontroller was also connected to a response button used to record participants’ reaction times to tactile stimulation.

Given known display latency in VR headsets^54–56^, we measured the display latency of our VR system using the photodiode-based method described by Le Chénéchal and Chatel-Goldman (2018)^56^. Specifically, in a pilot test of the VR setup, we used a photodiode to measure the average delay between issuing a command in Unity to present a visual stimulus and its actual appearance on the VR headset across 500 trials. This display latency was then directly compared with the execution latency of the microcontroller used to deliver tactile stimuli, also measured across 500 trials. The VR headset exhibited a mean display latency of 36 ms (*SD* < 1 ms), whereas the microcontroller’s execution latency was less than 1 ms (*SD* < 0.1 ms). Following the suggested correction procedure by Le Chénéchal and Chatel-Goldman (2018)^56^, a 36 ms delay in tactile stimulus delivery via the microcontroller was introduced to ensure temporal alignment between visual and tactile stimuli in the visuo-tactile task.

**Fig. 1:**
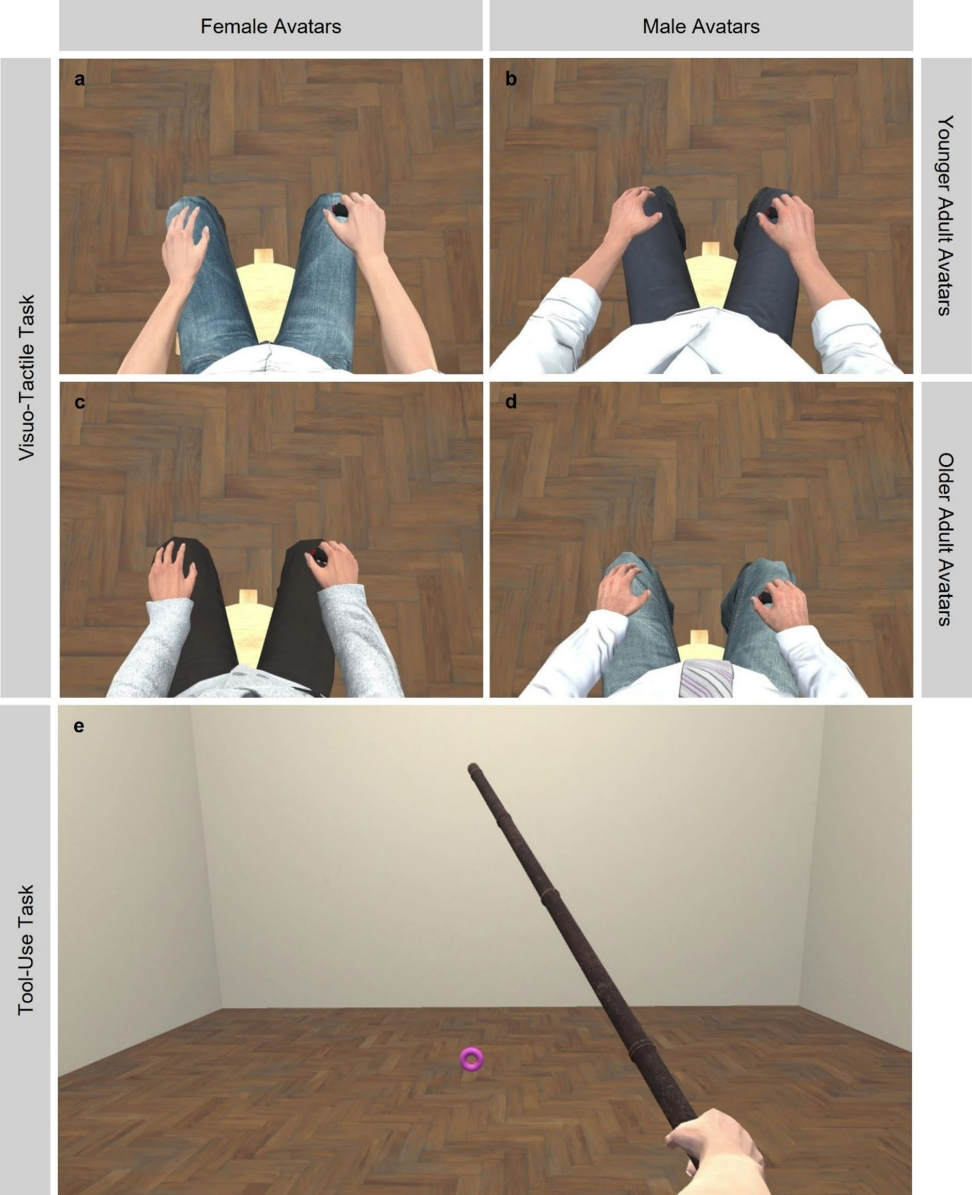
Virtual environment and avatars. Participants viewed a virtual room with beige walls and a wooden floor. In the visuo-tactile task, they saw an avatar matched for age and gender from a first-person perspective that was either a younger adult female (**a**), younger adult male (**b**), older adult female (**c**), or older adult male (**d**). In the tool-use task, the same matched avatar was shown (in this case a younger adult female avatar), however, the avatar’s right hand held a stick (replacing the response button) and was animated to mirror the participant’s right-arm movements (**e**). Participants used the controller held in their right hand to control the movement of the avatar’s arm to retrieve the pink ring with the stick.

The virtual setting of the experiment consisted of a 6 m × 7 m empty room with beige walls and a wooden floor. Participants viewed the scene from a first-person perspective that was co-located with an age and gender matched avatar selected from the Microsoft Rocketbox avatar library^57^. During the visuo-tactile task (see Visuo-tactile task subsection), the avatar appeared seated and static, holding a virtual model of the response button in its right hand (see Fig. 1a–d). In the tool-use task (see Tool-use task subsection), the avatar instead held a virtual stick that moved in synchrony with the VR controller participants held in their right hand (see Fig. 1e).

### Visuo-tactile task

To investigate how tool use affects PPS, a visuo-tactile task was employed before and after tool use. During the visuo-tactile task, participants held a response button in their right hand, while viewing the avatar in VR holding a virtual model of the same response button in its right hand (see Fig. 1a–d). Participants were instructed to respond as quickly as possible to tactile stimuli delivered to the back of their right hand. These tactile stimuli were presented either alone or alongside a task-irrelevant visual stimulus in the VR scene, consisting of a green ball 8 cm in diameter. Each trial began with a red fixation cross displayed on the ground for 1000 ms, followed by a randomly jittered delay ranging from 1000 to 1500 ms. After this delay, one of three trial types commenced.

In visuo-tactile trials, the visual stimulus first appeared on its own for 400 ms before an additional 100 ms in which both visual and tactile stimuli were presented simultaneously. Following this, participants had a 1000 ms window to respond. Unimodal tactile trials maintained the same timing structure as visuo-tactile trials but omitted the visual stimulus. Similarly, catch trials mirrored this timing structure but omitted the tactile stimulus. Since no tactile stimulus was presented in catch trials, participants were not expected to respond, and any responses were treated as false alarms to the visual stimulus. Consequently, the 1000 ms response window in catch trials was aligned with the onset of the visual stimulus.

For trials involving a visual stimulus, the visual stimulus was presented to the right of the visual midline in VR, centered on the avatar’s right hand. Crucially, the participant’s viewpoint was co-located with the avatar body, ensuring that the perceived distance of the visual stimulus from the avatar would be the same regardless of participant. The visual stimulus appeared 20 cm behind one of six target distances from the participant’s viewpoint and loomed toward the avatar’s right hand at a constant speed of 50 cm/s for 400 ms. Once the visual stimulus reached the target distance (target distances: 0.3 m, 0.7 m, 1.1 m, 1.5 m, 1.9 m, 2.3 m), it continued looming for another 100 ms concurrently with tactile stimulus presentation (in visuo-tactile trials) or alone (in catch trials).

**Fig. 2:**
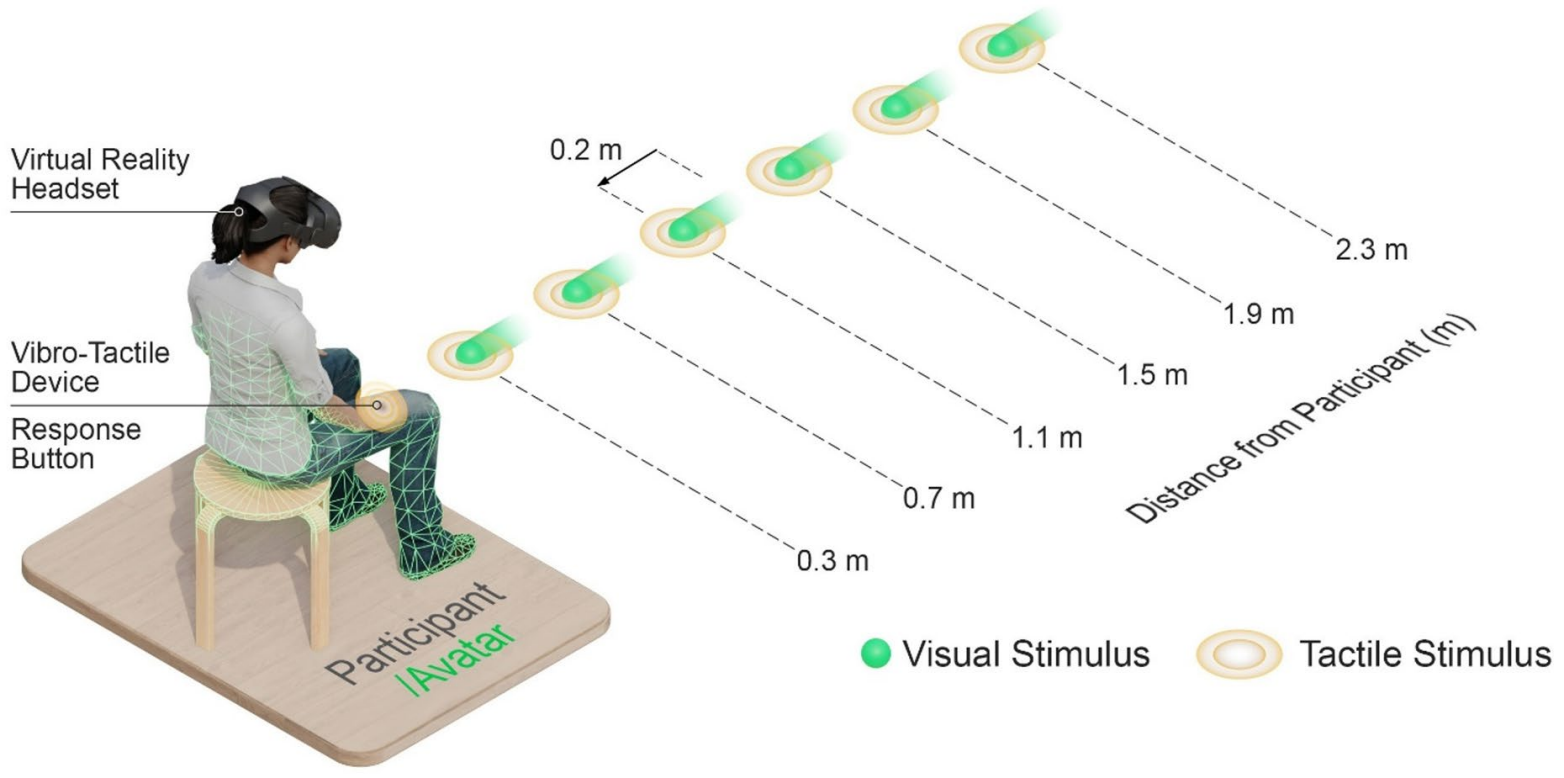
Visualization of the visuo-tactile task in virtual reality. Participants viewed a virtual scene through a virtual reality headset in which an avatar was co-located with their real body. A visual stimulus appeared shortly behind (0.2 m) one of six target distances from the participant’s viewpoint and loomed toward the avatar. When the visual stimulus reached the target distance, a tactile stimulus was delivered to the back of the participant’s right hand. Participants were then required to make a speeded response to the tactile stimulus by pressing a response button. Beyond these visuo-tactile trials, there were also unimodal tactile trials, in which only the tactile stimulus was presented, and catch trials in which only the visual stimulus was presented. As catch trials did not include a tactile stimulus, responses in these trials were considered as false alarms.

This “mini loom” design is a methodological adaptation of the classic visuo-tactile task. In the classic version, the visual stimulus always appears at the farthest location from the participant and looms toward them, with the timing of tactile stimulus presentation depending on the target distance (e.g., ^58–61^). A limitation of this approach is that the longer the visual stimulus looms for, the more participants anticipate the upcoming tactile stimulus, creating a confound known as temporal expectancy^62^. In the “mini loom” design, the visual stimulus instead appears at a location fairly close to (in the present study 20 cm behind) the target distance and looms toward the participant (or avatar in this case)^44–46^. As a result, the visual stimulus looms for the same duration before tactile stimulus presentation regardless of the target distance (see Fig. 2). By standardizing the duration that the visual stimulus looms for, the “mini loom” design controls for temporal expectancy and allows spatial effects to be assessed without this confound^62^.

The speed of the looming visual stimulus was informed by a previous VR study employing a visuo-tactile task^18^ and by evidence showing that the velocity of approaching stimuli can influence PPS estimates (e.g.,^2,60,63^). For example, Noel et al. (2018)^63^ demonstrated that PPS expands when stimuli approach at faster speeds (75 cm/s compared to 25 cm/s). To ensure that tool-use effects in far space would be clearly visible and not confounded by a velocity-induced expansion of PPS, we selected a stimulus speed of 50 cm/s.

### Tool-use task

During the tool-use task, participants held a VR controller in their right hand, while viewing the avatar in VR holding a virtual stick of 1.3 m length in its right hand (see Fig. 1e). When a participant moved the controller, the avatar’s arm along with the stick would follow the movement. This animation of the avatar’s arm through tracking of the VR controller was implemented using inverse kinematics^31^ as implemented in the Animation Rigging package (version 1.0.3) from Unity Technologies. This allowed participants to feel as if their own arm movements and the movement of the avatar’s arm were the same.

**Fig. 3:**
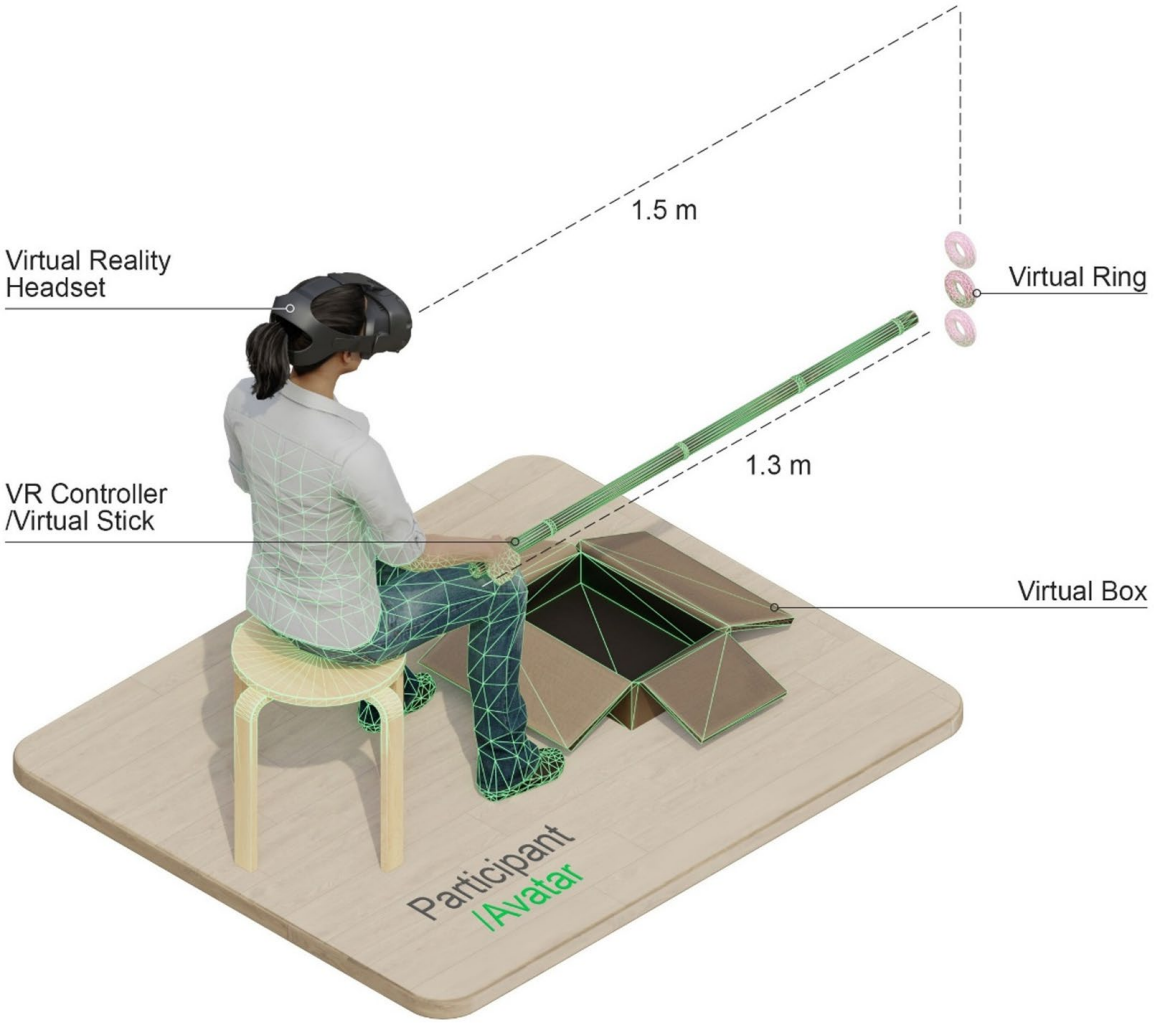
Visualization of the tool-use task in virtual reality. Participants held a virtual reality controller in their right hand, while being presented with a virtual scene through a virtual reality headset. Participants saw an avatar body co-located with their real body and holding a virtual stick of 1.3 m length. When participants moved the controller, the avatar’s arm and the virtual stick would follow the movement. A virtual pink ring would appear at one of three possible heights at 1.5 m from the participant’s viewpoint. Participants were required to use the virtual stick to move the virtual ring from far into near virtual space and put the virtual ring in a box on the ground. Once participants had done this the virtual ring would disappear, and a new virtual ring would appear at one of the three possible heights. This was repeated a total of 150 times.

In this context participants were required to move the end of the stick into a pink ring displayed 1.5 m in front of them at a height of either 0.55 m, 0.65 m, or 0.75 m from the ground in the virtual scene (see Fig. 3). Once the end of the stick reached the center of the ring, the controller would vibrate, indicating that the ring was now attached to the stick. At this point participants had to move the ring into a box placed in front of the avatar. As soon as the ring was in the box it would disappear, and a new ring would appear at one of the three possible heights.

The distance of 1.5 m for the rings was based on the distance of the objects used in Petrizzo et al. (2024)^27^, while the length of the stick was based on pilot testing showing that a stick of 1.3 m allowed participants to comfortably reach a distance of 1.5 m in the VR environment when extending their arm, as well as comfortably retract their arm to put the rings in the box at the avatar’s feet.

### Avatar ownership scores

To compare avatar ownership before and after tool use, participants completed the standardized Embodiment Questionnaire^47^. The questionnaire items were translated into German and independently validated by two native German speakers. Participants rated each item on a 7-point Likert scale (1 = strongly disagree, 7 = strongly agree). Our analysis focused on the six items comprising the ownership subscale. Following the approach suggested by Peck and Gonzalez-Franco (2021)^47^, we calculated the ownership score as the mean of the six items. The English and German versions of the questionnaire items are provided in Table S1 and S2 of the Supplementary Materials respectively, along with an analysis of internal consistency for the translated German version.

### Procedure

Participants first completed the Edinburgh Handedness Inventory^52^ to establish right-handedness (cutoff was set at a laterality quotient of + 60), followed by a tactile threshold test to measure their tactile threshold. During this test a tactile stimulus was presented on the back of the participant’s right hand in the same location as tactile stimulus presentation during the visuo-tactile task. The tactile stimulus started at 5% intensity and increased in intensity in steps of 5% until 100% intensity was reached. After each intensity increase, participants were asked if they perceived a stimulus and the first intensity at which the participant gave three positive detection responses was taken as the participant’s tactile threshold. After this, participants completed a short questionnaire about their gaming and VR experience (see Table S3 and S4 of the Supplementary Materials for the German and English versions of this questionnaire respectively).

Participants were then sat on a stool and equipped with a VR headset. They were then guided to bring their body into the posture the avatar would have, after which the virtual scene was started and centered so that participants saw the avatar body from a first-person perspective overlapping with their physical body. To familiarize participants with the virtual setup, they were then asked to briefly visually explore the virtual room and avatar, while keeping their posture matched with the avatar’s. After this familiarization period, they completed a short practice session of the visuo-tactile task, followed directly by an experimental block of the visuo-tactile task to provide a baseline assessment of PPS before tool use. At the end of this block the VR headset was removed, and participants were required to respond to the Embodiment Questionnaire. After a short break the VR headset was placed back on the participant’s head and participants completed the tool-use task. This was followed by another block of the visuo-tactile task to provide a measurement of PPS after tool use. The VR headset was then removed again and participants responded to the Embodiment Questionnaire once more.

Both blocks of the visuo-tactile task consisted of 180 trials. These 180 trials were presented in randomized order and consisted of 120 visuo-tactile trials (20 per distance level), 30 unimodal tactile trials, and 30 catch trials (5 per distance level). For the tool-use task there were a total of 150 trials, with the ring being displayed at each of the three possible heights 50 times in a randomized order.

### Data preprocessing and analysis

Data analyses were conducted using R Statistical Software (version 4.3.1; R Core Team, 2023). For the visuo-tactile task, unimodal tactile and visuo-tactile trials with reaction times below 100 ms or in which no response was given (response cut off at 1000 ms) were excluded. Additionally, unimodal tactile and visuo-tactile trials with reaction times exceeding ± 3 standard deviations from each participant’s block mean were identified as outliers and removed. On average, these filters resulted in the exclusion of 3.1% of trials for YAs and 2.9% for OAs.

Overall performance in the visuo-tactile task was high, with mean false alarm rates in catch trials of 2.9% for YAs and 0.7% for OAs. In the tool-use task, there was a significant difference between YAs and OAs in the mean duration of tool-use trials (*W* = 131, *p* < .0001, *r* = .55), with OAs (*M* = 3.46 s, *SD* = 0.77 s) taking on average 0.78 s longer than YAs (*M* = 2.68 s, *SD* = 0.60 s) to complete a trial. This aligns with a general slowing of movement in older compared to younger adults^64,65^. However, crucially, all participants completed the full set of 150 tool-use trials.

The degree of multisensory facilitation in the visuo-tactile task was measured using the reaction time facilitation (RTF) of responses to the tactile stimulus through concurrent presentation of the visual stimulus. To calculate RTF, reaction times during unimodal tactile trials are subtracted from those during visuo-tactile trials^18,28,29,60^. A negative RTF therefore indicates multisensory facilitation, meaning faster responses in visuo-tactile trials than in unimodal tactile ones. We computed each participant’s mean unimodal tactile reaction time at both measurement time points (before and after tool use), as well as their mean visuo-tactile reaction time at both time points and each visual stimulus distance. Based on this, each participant’s RTF was calculated at the two time points and at each visual stimulus distance. By performing this calculation at each time point separately, we controlled the resulting RTF values for potential unspecific learning effects, as these would be expected to affect both unimodal tactile and visuo-tactile trials.

To assess RTF, we first conducted a general ANOVA including the between-subject factor age and the within-subject factors time point and distance. A significant three-way interaction between time point, distance, and age in this model constituted the prerequisite for conducting ANOVAs separately within each age group. For the general ANOVA, homogeneity of variance for the between-subject age factor was examined using Levene’s test at each level of the within-subject factors time point and distance, with all tests indicating non-significant differences (all *p* > .05). For all three ANOVAs, sphericity for the within subject factors time point and distance was assessed using Mauchly’s test. Where violations of sphericity were detected, the Greenhouse-Geisser correction was applied. Normality of residuals for each ANOVA model was evaluated through visual inspection of QQ-plots and frequency histograms, with no notable deviations observed. All post-hoc tests of RTF were two-tailed and adjusted for multiple comparisons using the Holm-Bonferroni method. Adjusted p-values are denoted with the subscript *adjusted* in the Results section.

Avatar ownership was measured as the average of six Likert-scale items. While Likert-scale items are ordinal, averaging across several items produces a composite score that can be reasonably treated as interval scaled^66,67^. To maintain a conservative approach, we nevertheless applied non-parametric tests (Wilcoxon signed-rank and rank-sum tests) for comparisons involving avatar ownership. For the correlation analysis, we assessed whether changes in ownership (calculated as the difference between ownership scores after tool use and before tool use) were associated with changes in overall RTF (calculated as the difference between overall RTF after tool use and before tool use). We employed Spearman’s rank correlation for this analysis, which relies on the rank ordering of change scores rather than their exact values. This again provides a more conservative approach and allows to account for potential non-linearity in the data.

## Results

### Tool use and PPS measured by multisensory reaction time facilitation

To examine if the effect of tool use on the spatial distribution of multisensory facilitation differed between age groups, we conducted a three-way ANOVA on RTF with time point (two levels: before tool use, after tool use) and distance (six levels: 0.3 m, 0.7 m, 1.1 m, 1.5 m, 1.9 m, 2.3 m) as within-subject factors, and age (two levels: younger, older) as a between-subject factor. We found significant main effects of time point (*F*(1, 52) = 19.01, *p* < .0001, *η*_*p*_^*2*^ = 0.27) and distance (*F*(2.03, 105.34) = 30.40, *p* < .0001, *η*_*p*_^*2*^ = 0.37). Importantly, there was also a significant three-way interaction between time point, distance, and age (*F*(4.18, 217.14) = 2.48, *p* = .042, *η*_*p*_^*2*^ = 0.05), indicating that the effect of time point on RTF across distances differed between age groups. This suggests an age-dependent effect of VR tool use on the spatial distribution of multisensory facilitation.

**Fig. 4:**
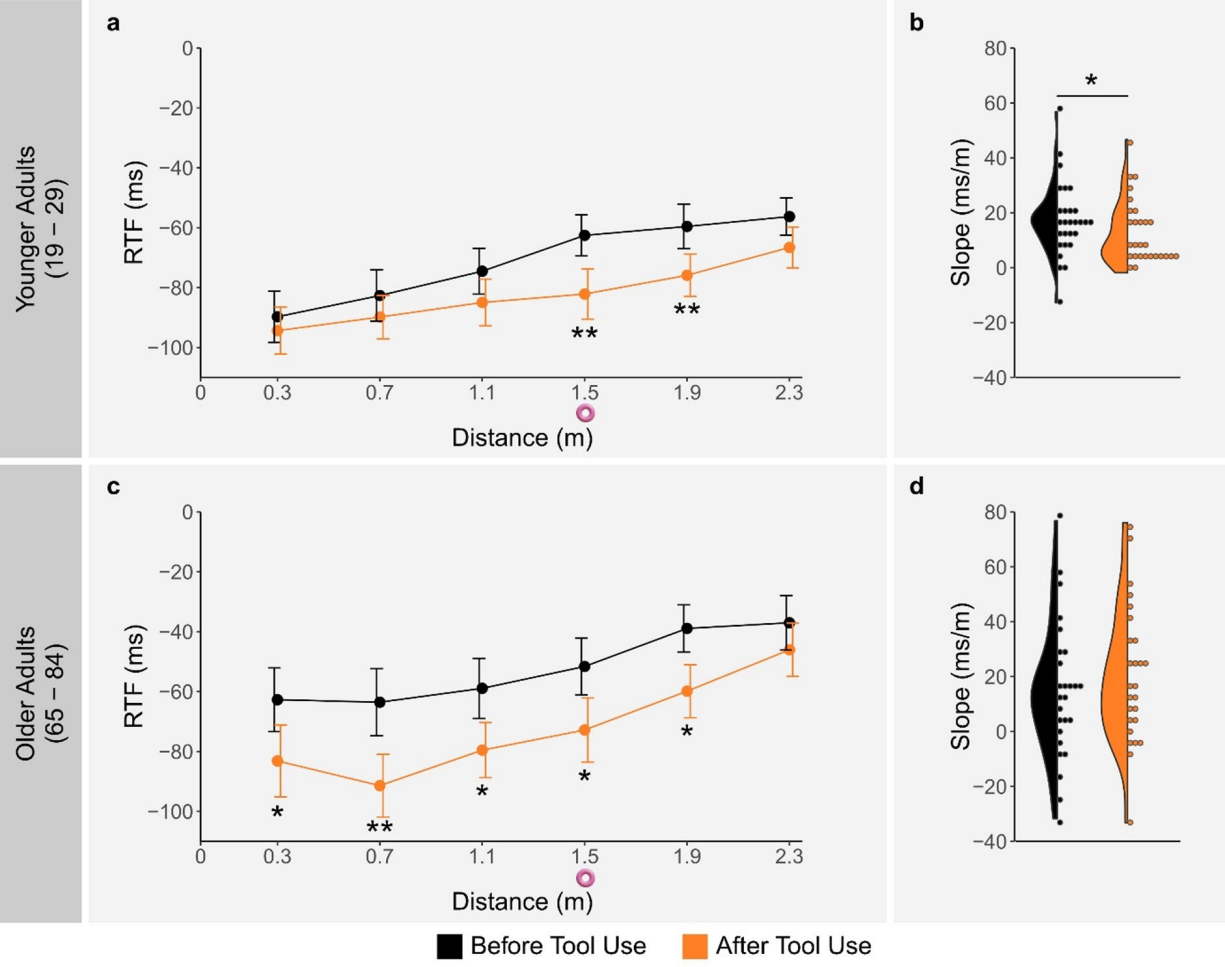
Reaction time facilitation (RTF) results in younger adults (YAs) and older adults (OAs). RTF is shown as a function of time point and distance in YAs (**a**) and OAs (**c**), with negative values indicating faster responses in visuo-tactile trials compared to unimodal tactile trials, error bars representing ± 1 standard error of the mean, and the location of tool use denoted on the x-axis with a pink ring. Violin and dot plots show the distribution of individual RTF-over-distance slopes (ms/m) at both time points in YAs (**b**) and OAs (**d**). For all plots, asterisks indicate statistically significant differences between time points.

The three-way interaction was followed up with age-specific analyses by conducting separate two-way ANOVAs on RTF for each age group, with time point and distance as within-subject factors. In YAs, we found significant main effects of time point (*F*(1, 27) = 11.03, *p* = .003, *η*_*p*_^*2*^ = 0.29) and distance (*F*(2.43, 65.66) = 29.75, *p* < .0001, *η*_*p*_^*2*^ = 0.52), along with a significant interaction between time point and distance (*F*(5, 135) = 3.73, *p* = .003, *η*_*p*_^*2*^ = 0.12). This indicates that the distance effect differed before and after tool use. Post-hoc comparisons of RTF before and after tool use at each distance level revealed significantly stronger RTF after tool use at 1.5 m (*t*(27) = 4.05, *p*_*adjusted*_ = .002, *d* = 0.76) and 1.9 m (*t*(27) = 3.53, *p*_*adjusted*_ = .008, *d* = 0.67), while this comparison was not significant at the other distance levels (all *p*_*adjusted*_ > .05). Given that stronger RTF was observed at and close to the location where tool use occurred (1.5 m and 1.9 m), this suggests that VR tool use in YAs led to a location-specific increase in multisensory facilitation (see Fig. 4a).

In OAs, although there were also significant main effects of time point (*F*(1, 25) = 9.47, *p* = .005, *η*_*p*_^*2*^ = 0.28) and distance (*F*(1.84, 45.98) = 11.40, *p* < .001, *η*_*p*_^*2*^ = 0.31), the interaction between time point and distance was not significant (*F*(5, 125) = 1.71, *p* = .138, *η*_*p*_^*2*^ = 0.06). This indicates that while there was a decrease in RTF with distance both before and after tool use, as well as overall stronger RTF after tool use, the effect of distance on RTF did not differ between time points. Post-hoc comparisons of RTF before and after tool use at each distance level showed significantly stronger RTF after tool use at 0.3 m (*t*(25) = 3.03, *p*_*adjusted*_ = .019, *d* = 0.59), 0.7 m (*t*(25) = 4.06, *p*_*adjusted*_ = .003, *d* = 0.80), 1.1 m (*t*(25) = 3.09, *p*_*adjusted*_ = .019, *d* = 0.61), 1.5 m (*t*(25) = 2.55, *p*_*adjusted*_ = .034, *d* = 0.50), and 1.9 m (*t*(25) = 3.34, *p*_*adjusted*_ = .013, *d* = 0.66), but not at 2.3 m (*p*_*adjusted*_ > .05). This pattern in OAs suggests a location-nonspecific increase in multisensory facilitation after VR tool use, rather than a location-specific effect (see Fig. 4c).

To assess whether the observed three-way interaction between time point, distance, and age, as well as the age-specific effects of time point and distance might be influenced by other age-related differences, we conducted additional covariate analyses, which are reported in the Supplementary Materials. Specifically, we examined potential age differences in gaming and VR experience, tactile sensitivity thresholds, and tool-use performance metrics based on tool-use trial duration. While average weekly gaming time and changes in tool-use trial duration between the first and second half of trials did not differ between age groups, age-related differences were observed in tactile sensitivity, total time using VR, and mean tool-use trial duration. These factors were therefore included as covariates in additional control analyses, with results showing that inclusion of these covariates did not yield significant effects and did not alter the significance of the three-way interaction or subsequent age-specific effects. We therefore reported the primary analyses without covariates here.

Relatedly, to establish whether there were age-related differences in baseline multisensory facilitation, we conducted a two-way ANOVA on RTF before tool use with distance as a within-subject factor and age as a between-subject factor. This analysis revealed no significant main effect of age (*F*(1, 52) = 2.80, *p* = .101, *η*_*p*_^*2*^ = 0.05) and no interaction between age and distance (*F*(2.55, 132.34) = 0.97, *p* = .397, *η*_*p*_^*2*^ = 0.02), with the only significant effect being a main effect of distance (*F*(2.55, 132.34) = 20.88, *p* < .0001, *η*_*p*_^*2*^ = 0.29). This indicates that the observed differences in the effect of tool use on multisensory facilitation between younger and older adults are not attributable to baseline differences between age groups.

To further highlight the observed age-related difference in the effect of VR tool use on the spatial distribution of multisensory facilitation, we fitted simple linear models to each participant’s RTF over distance both before and after tool use and compared the resulting slopes. Among YAs, this comparison revealed a significant change, with steeper positive slopes before tool use (*M* = 17.74, *SD* = 13.61) than after (*M* = 13.11, *SD* = 11.80; *V* = 113, *p* = .040, *r* = .39). This indicates a reduced rate of decline in RTF over distance following tool use, consistent with a change in the spatial distribution of multisensory facilitation. In contrast, OAs showed no significant change in slope (before tool use: *M* = 14.97, *SD* = 25.01; after tool use: *M* = 20.49, *SD* = 24.94; *V* = 215, *p* = .328, *r* = .20). These results suggest that, while VR tool use in YAs affected the spatial distribution of multisensory facilitation (see Fig. 4b), in OAs it primarily increased the overall degree of multisensory facilitation, without affecting its spatial distribution (see Fig. 4d).

### Tool use and avatar ownership

**Fig. 5:**
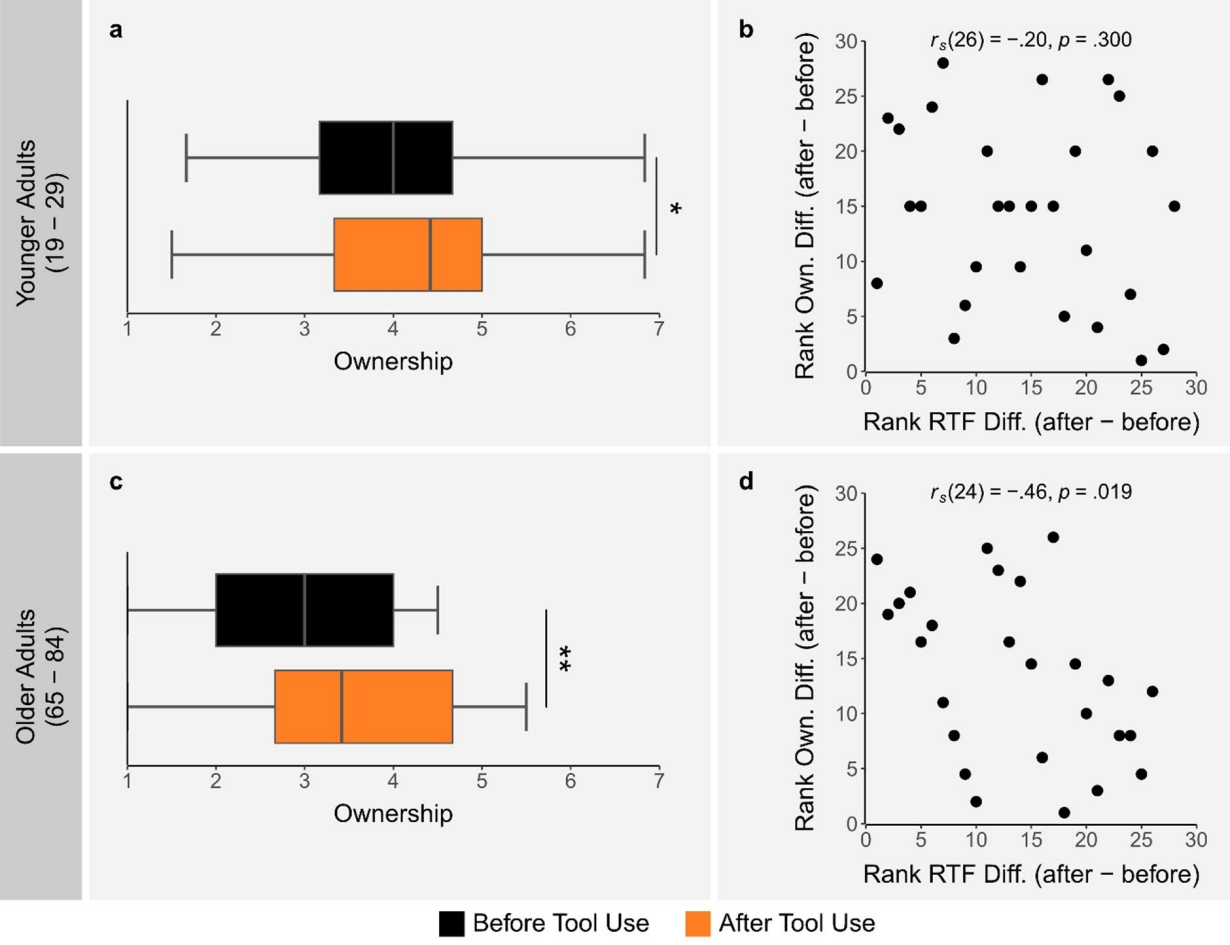
Avatar ownership score results in younger adults (YAs) and older adults (OAs). Box and whisker plots show the ownership scores at both time points in YAs (**a**) and OAs (**c**). In these box and whisker plots, the center line represents the median, the box spans the first to third quartiles, and the whiskers extend to the smallest and largest value within 1.5 times the interquartile range below the first quartile and above the third quartile respectively. Asterisks indicate statistically significant differences between time points for the ownership plots. Scatterplots show the Spearman correlation between the ranked ownership differences (ownership after tool use − ownership before tool use) and the ranked overall RTF differences (overall RTF after tool use − overall RTF before tool use) in YAs (**b**) and OAs (**d**).

To examine how VR tool use affected perceived ownership over the virtual avatar, we compared avatar ownership scores before and after tool use. Wilcoxon signed-rank tests revealed significantly greater ownership scores after compared to before tool use for both YAs (*V* = 257.5, *p* = .038, *r* = .40) and OAs (*V* = 237, *p* = .003, *r* = .58). Moreover, to compare ownership scores between age groups, Wilcoxon rank-sum tests were applied. This revealed that ownership scores before tool use were significantly lower for OAs compared to YAs (*W* = 532, *p* = .004, *r* = .40), but only marginally lower for OAs compared to YAs after tool use (*W* = 464, *p* = .084, *r* = .24) and not significantly different when comparing after tool use in OAs to before tool use in YAs (*W* = 416, *p* = .372, *r* = .12). These results suggest that VR tool use led to increased avatar ownership in both YAs (see Fig. 5a) and OAs (see Fig. 5c). Furthermore, although OAs experienced weaker avatar ownership compared to YAs before tool use, after tool use their ownership levels were comparable to those of YAs.

As an exploratory analysis, we investigated whether the effect of tool use on avatar ownership scores was associated with the effect of tool use on RTF. To do this, we calculated each participant’s difference in ownership score between time points (ownership after tool use − ownership before tool use), as well as their difference in overall RTF (i.e., the mean RTF across all distances) between time points (overall RTF after tool use − overall RTF before tool use). We then ran a Spearman’s correlation between the rank of these differences separately for YAs and OAs. In OAs, we found a moderate and significant negative correlation (*r*_*s*_(24) = − 0.46, *p* = .019; see Fig. 5d), indicating that OAs who reported a greater increase in avatar ownership following tool use also had a greater increase in overall RTF (i.e., a more negative overall RTF, indicating stronger multisensory facilitation). While this correlation went in the same direction in YAs, it was weak and not significant (*r*_*s*_(26) = − 0.20, *p* = .300; see Fig. 5b).

## Discussion

We investigated whether tool use in VR affects the representation of PPS, and whether this effect differs between younger and older adults. In addition, we examined whether VR tool use affects the sense of ownership over a virtual avatar and explored whether this is related to changes in PPS. In YAs, tool use in VR led to a change in the spatial distribution of multisensory facilitation, with results suggesting a location-specific modulation of PPS. In contrast, in OAs there was no change in the spatial distribution of multisensory facilitation, with results suggesting a location-nonspecific, rather than a location-specific modulation of PPS after VR tool use. Interestingly, both age groups reported an enhanced sense of ownership over the virtual avatar following VR tool use. In OAs, this increase in ownership was positively associated with the overall increase in multisensory facilitation, whereas this relationship was not significant in YAs.

The change in the spatial distribution of multisensory facilitation following tool use in YAs, further substantiates the notion that VR-based tool use can affect PPS representations and supports the findings of Petrizzo et al. (2024)^27^. This result offers insight into the plasticity of PPS to immersive virtual environments. Given the proposed role of PPS in guiding interactions with the environment^5,8^, such modulation raises the possibility that skills practiced in VR environments might relate to similar behaviors in real-world contexts. This potential has implications in areas such as VR-based interventions for skill training and rehabilitation. Nonetheless, whether VR-induced changes in PPS may transfer as measurable improvements in real-world performance remains an open question and should be directly tested in future studies.

A noteworthy result in YAs, is that post-hoc tests revealed a significant difference in multisensory facilitation at the tool-use location (1.5 m), but also at the next farthest distance (1.9 m), suggesting that tool use may have modulated PPS beyond the immediate area of interaction. Interestingly, no significant effect was observed at the closer 1.1 m distance, despite it being equidistant from 1.5 m. This asymmetry may be explained by participants being able to reach beyond 1.5 m with the virtual stick, potentially affording an extended action space that biased PPS modulation toward farther distances. Additionally, the task emphasized forward extension of the virtual stick through rings, further highlighting far space over near.

It is important to note that while the results in YAs suggest a change in the spatial distribution of PPS after tool use, we did not directly assess if there was an extension of the boundary of PPS. A common method used to estimate the PPS boundary in previous studies involves identifying the distance at which reaction times in visuo-tactile trials are no longer significantly different from the average reaction time in unimodal tactile trials (e.g.,^12,58,60^). However, in our study, RTF remained significantly different from zero (i.e., the average reaction time in unimodal tactile trials) at all tested distances for both YAs and OAs, indicating that the PPS boundary extended beyond the range of distances included.

Notably, this pattern of RTF being significantly different from zero at all distances, has also been reported in other studies employing the adapted version of the visuo-tactile task utilizing a “mini loom” design (e.g.,^29,45,46^). This recurring pattern suggests that controlling temporal expectancy effects^62^ with this version of the visuo-tactile task constrains conventional PPS boundary inference while still capturing relative spatial effects. Therefore, future research should further investigate how different versions of the visuo-tactile task influence the measurement of PPS, particularly to distinguish effects driven by temporal expectancy from those that genuinely reflect changes in the spatial representation of PPS.

The stable spatial distribution of multisensory facilitation following tool use in OAs suggests that aging alters the expected modulation of PPS through tool use. This aligns with previous studies in physical settings showing that, unlike younger adults, older adults do not exhibit changes in their perceived distance to targets following tool use^36–38^. However, although the spatial distribution of multisensory facilitation did not change in OAs, they did exhibit a pattern suggesting a location-nonspecific increase in multisensory facilitation following tool use. This may indicate that there was a general modulation of PPS following tool use in OAs.

Related to this, OAs exhibited a significant correlation between the increase in overall multisensory facilitation and the increase in avatar ownership. This association could help explain the suggested general modulation of PPS in OAs, given that prior research has linked perceived avatar ownership to modulations of PPS^7,18,28^. It may be that the heightened sense of owning a virtual body contributed to a general enhancement of PPS around that virtual body. Thus, the suggested location-nonspecific increase in multisensory facilitation in OAs may have reflected a general modulation of PPS related to avatar ownership rather than tool use specifically.

This correlation in OAs raises the question of why the same correlation was weak and non-significant in YAs, despite YAs also demonstrating increased ownership following tool use. One potential explanation lies in the baseline differences: YAs reported significantly higher ownership before tool use compared to OAs, while ownership levels were comparable between age groups after tool use. Moreover, the effect of tool use on ownership scores had a medium effect size in YAs (*r* = .40), whereas the effect size was large for OAs (*r* = .58). Thus, the relative increase in ownership for YAs may have had a diminished or negligible impact on overall multisensory facilitation within the VR environment.

These considerations exemplify the importance of incorporating realistic full or partial virtual body representations when studying PPS in VR. They may also have implications for the design of VR applications. If greater avatar ownership is linked to enhanced PPS representation in VR, establishing a strong sense of ownership before VR training or rehabilitation interventions could potentially support their efficacy. However, it is important to emphasize that the analysis underlying this observed relationship in OAs was exploratory and correlational. Further research is required to validate these preliminary findings, as well as to establish a causal relation between the sense of avatar ownership and the representation of PPS within VR.

The observed increase in ownership following tool use is itself of interest, as it further confirms that animating avatars to follow participants’ movements can effectively enhance perceived avatar ownership^30^. This finding is of particular interest in OAs, given our recent findings suggesting that older adults are less susceptible than younger adults to a third-person full-body illusion, in which participants are expected to experience ownership over an avatar viewed from a third-person perspective in VR^29^. In the present study, while ownership was initially weaker in OAs compared to YAs, comparable ownership scores were observed between these groups after tool use. This suggests that, under the right circumstances, it is possible for older adults to experience levels of avatar ownership in VR that are comparable to those in younger adults. Future research should examine whether incorporating synchronous movement, rather than only synchronous visuo-tactile stimulation, could also improve induction of the third-person full-body illusion in older adults.

An important consideration in interpreting our findings concerns the potential of unspecific effects (e.g., practice effects) from repeated exposure to the visuo-tactile task and whether they could have influenced the general increase in multisensory facilitation observed in OAs. Here it is important to note that the RTF measure is calculated as the difference between reaction times in unimodal tactile and visuo-tactile trials and was computed separately for each individual and in each block of the visuo-tactile task. Because unspecific effects would be expected to influence both unimodal tactile and visuo-tactile trials, such effects would cancel out in the difference score. RTF should therefore be relatively insensitive to unspecific effects, making it unlikely that they account for the observed increase in multisensory facilitation in OAs.

One general point related to the interpretation of tool-use effects on PPS, is that while an increase in multisensory integration in the area of tool use is generally interpreted as a remapping of PPS (e.g.,^8,14,15,33,34^), an alternative account based on a shift in visual attention to the functional end of the tool has also been suggested^68,69^. However, given that our results indicated significant differences at multiple distances (1.5 m and 1.9 m in YAs and all distances except the farthest in OAs), an explanation through a shift in visual attention to the tip of the virtual stick does not appear to be in line with this pattern of results.

A limitation of the study relates to the extent of motion tracking used during the tool-use task. We used controller-based tracking combined with inverse kinematics^31^ to animate the right arm of the avatar to follow participants’ arm movements. This approach provides less precise tracking than with additional trackers placed on the arm or full motion-capture suits. However, a recent study showed that controller-driven inverse kinematics used to animate the upper body of avatars can lead to strong perceived avatar embodiment and produce comparable (and at times even preferrable) user-experience results to full motion-tracking of the arm^32^. Consistent with this evidence, avatar ownership ratings in the present study increased after tool use in both age groups, indicating that the controller-driven tracking employed was sufficient to enhance the sense of ownership. Moreover, full body tracking, including the legs, would have offered limited additional benefit in our context, as participants remained seated throughout the experiment.

The extent of motion tracking would have also been relevant as a movement-related performance metric during the tool-use task. More detailed tracking information would have provided additional insight into how movement-related variables, such as movement accuracy and trajectory, may have influenced the observed effects on PPS. Such measures would have allowed a more direct assessment of the relationship between the sensorimotor processes of tool use and age-related differences in PPS modulation. Although the inclusion of mean tool-use trial duration as a covariate did not alter the age-related effects in our analyses, and we additionally observed a comparable reduction in mean tool-use trial duration over time across age groups (see Supplementary Materials), more fine-grained movement-related performance metrics would have provided a more direct assessment of the contribution of sensorimotor processes to PPS modulation. Related to this, a limitation of the present study is that we did not include a control condition with no tool use or a sham action. Including such a control condition in future studies would help further strengthen the causal inferences regarding the specific contribution of tool use to PPS modulation.

Another limitation concerns the simplicity of the virtual tool used in the present study. While employing a virtual stick allowed us to investigate the effects of using rudimentary tools in VR, little is known about how the modulation of PPS may differ following the use of more complex tools. Although Petrizzo et al. (2024)^27^ examined the effect of VR tools involving different motor routines, this should be further expanded upon. VR provides an ideal context for systematically varying tool complexity while maintaining experimental control. Future studies should take advantage of this to systematically examine whether the adaptation of PPS following tool use may depend on the functional demands or structure of the tool, as well as the environment in which the tool use takes place. Such investigations would not only deepen our understanding of how PPS adapts to different classes of tools but would also have direct implications for the design of VR-based training and rehabilitation interventions, where more ecologically valid and task-specific tools may be required.

A further limitation is that we relied on a subjective measure of avatar ownership. More objective physiological measures have been used as indicators of ownership in other studies, including increased galvanic skin response to threats approaching the avatar or a decrease in skin temperature during body illusions^70^. Including such measures would have strengthened the scope of the ownership assessment and could have provided converging evidence for the relationship between ownership and multisensory facilitation observed in OAs. Future studies would benefit from combining subjective reports with such physiological measures.

## Conclusion

This study provides further evidence of how tool use in VR affects PPS, revealing age-related differences in this effect. In YAs, the pattern of results suggests a location-specific modulation of PPS after tool use. In contrast, OAs did not show a location-specific modulation but did exhibit results suggesting a general modulation of PPS. Notably, the increase in overall multisensory facilitation in OAs was linked to their increased sense of avatar ownership, raising the possibility that the heightened sense of owning a virtual body following tool use contributed to an enhanced representation of PPS within the VR environment. However, this interpretation remains speculative and warrants further investigation. Overall, these findings suggest that PPS remains responsive to VR environments in older age, but that there are age-related differences in the mechanisms underlying the modulation of PPS through tool use in VR. While limitations remain and effects need to be examined in more complex VR contexts, these insights have implications for the development of age-inclusive VR applications and interventions, as well as for advancing the theoretical understanding of PPS.

## Supplementary Information

Below is the link to the electronic supplementary material.

Supplementary Material 1

## Data Availability

The datasets analyzed for the present study are available at the following Open Science Framework repository link: https://osf.io/e32yz/overview?view_only=5436b7872a734a9e830052fd5a0b869e.
