# Supplementary material for: Adult age differences in the modulation of peripersonal space after tool use in virtual reality
Source: Sci Rep. 2026 Feb 23;16:7505. doi: 10.1038/s41598-026-41116-y (PMC12932838; doi:10.1038/s41598-026-41116-y)
Supplement: Supplementary file 1 — Supplementary Material 1 [file 41598_2026_41116_MOESM1_ESM.docx]

**Supplementary Materials**

**Adult age differences in the modulation of peripersonal space**

**after tool use in virtual reality**

Dariusz O’Leary^1,2,3,*^, Yichen Fan^1,3,4^, Jens Krzywinski^1,3,4^, & Shu-Chen Li^1,2,3,*^

1. 6G-life Research Hub, TU Dresden, Dresden, Germany
2. Chair of Lifespan Developmental Neuroscience, Faculty of Psychology, TU Dresden, Dresden, Germany
3. Centre for Tactile Internet with Human-in-the-Loop (CeTI), TU Dresden, Dresden, Germany
4. Chair of Industrial Design Engineering, TU Dresden, Dresden, Germany

^*^Corresponding authors: dariusz.oleary@tu-dresden.de / shu-chen.li@tu-dresden.de

**Covariate Analyses**

To assess whether observed age-related effects on RTF might be confounded by other age-related differences, we conducted additional analyses to identify potentially relevant covariates. Consistent with findings indicating a reduction in tactile sensitivity with age^1,2^, OAs had significantly higher log-transformed tactile thresholds compared to YAs (*t*(51.80) = 7.23, *p* < .0001, *d* = 1.97). Although there was no significant difference in average weekly gaming time between age groups (*W* = 424, *p* = .278, *r* = 0.15), YAs reported significantly greater total time spent using VR headsets compared to OAs (*W* = 562, *p* < .001, *r* = 0.49). Moreover, aligning with the general slowing of movement with age^3,4^, the mean trial duration in the tool-use task was significantly longer in OAs compared to YAs (*W* = 131, *p* < .0001, *r* = 0.55). As an indicator of tool-use performance over time, we also compared mean trial duration between the first and second half of the tool-use task within each age group. This revealed a significant reduction in trial duration in the second half for both YAs (*V* = 381, *p* < .0001, *r* = 0.77) and OAs (*V* = 292, *p* = .002, *r* = 0.58). Importantly, the magnitude of this reduction (mean trial duration in second half – mean trial duration in first half) did not differ significantly between age groups (*W* = 421, *p* = .331, *r* = 0.13), with YAs (*M* = −0.44 s, *SD* = 0.35 s) and OAs (*M* = −0.43 s, *SD* = 0.77 s) showing a comparable decrease in trial duration. This pattern suggests similar gains in sensorimotor efficiency over time across age groups.

Given that tactile threshold, total time using VR, and mean tool-use trial duration differed significantly between age groups, we next examined whether observed age-related effects might be confounded by these factors. We therefore repeated the three-way ANOVA on RTF including tactile threshold, total time using VR, and mean tool-use trial duration as covariates, to see whether these factors affected the age-related three-way interaction. Within this model, there was no significant effect of tactile threshold (*F_covariate_* (1, 49) = 2.69, *p_covariate_* = .108, *η_p_^2^_covariate_* = 0.05), total time using VR (*F_covariate_* (1, 49) = 0.07, *p_covariate_* = .798, *η_p_^2^_covariate_* = 0.001), or mean tool-use trial duration (*F_covariate_* (1, 49) = 2.17, *p_covariate_* = .148, *η_p_^2^_covariate_* = 0.04) on RTF. Moreover, the critical three-way interaction between time point, distance, and age remained significant (*F_covariate_*(5, 245) = 2.89, *p_covariate_* = .015, *η_p_^2^_covariate_* = 0.06) and showed a small increase in effect size (*η_p_^2^* = 0.05 vs. *η_p_^2^_covariate_* = 0.06). This indicates that the observed age-related three-way interaction is not attributable to age differences in tactile sensitivity, experience using VR, or the mean duration of tool-use trials.

In addition, we also checked whether the effects observed within each age group could have been influenced by these covariates. For this reason, we repeated the separate age group ANOVAs on RTF including tactile threshold, total time spent using VR, and mean tool-use trial duration as covariates. None of the covariates had a significant effect on RTF in either of these models (all p > .05). Moreover, the same pattern of results was found in these models compared to the models without covariates. In YAs there was a significant main effect of time point (*F_covariate_* (1, 24) = 10.52, *p_covariate_* = .003, *η_p_^2^_covariate_* = 0.31), and distance (*F_covariate_* (2.37, 56.92) = 28.66, *p_covariate_* < .0001, *η_p_^2^_covariate_* = 0.54), as well as a significant interaction between time point and distance (*F_covariate_* (5, 120) = 3.58, *p_covariate_* = .005, *η_p_^2^_covariate_* = 0.13). In OAs, the model showed a significant main effect of time point (*F_covariate_* (1, 22) = 9.39, *p_covariate_* = .006, *η_p_^2^_covariate_* = 0.30) and distance (*F_covariate_* (1.68, 36.99) = 10.99, *p_covariate_* < .001, *η_p_^2^_covariate_* = 0.33), but no significant interaction between time point and distance (*F_covariate_* (5, 110) = 1.80, *p_covariate_* = .118, *η_p_^2^_covariate_* = 0.08). Together, the results of these additional analyses indicate that the effects observed within age groups were not attributable to individual differences in tactile sensitivity, experience using VR, or the mean duration of tool-use trials.

**Internal Consistency of Translated German Version of Embodiment Questionnaire**

We calculated Cronbach’s α as a measure of internal consistency for the translated German version of the Embodiment Questionnaire (Q1 – Q14 in Table S2). This was done across participants at both time points (due to Cronbach’s α being time-specific) and resulted in Cronbach’s α values of 0.90 before tool use and 0.91 after tool use, indicating excellent internal consistency. We did the same for the ownership subscale specifically (Q4, Q8 – Q12 in Table S2) and found Cronbach’s α values of 0.80 before tool use and 0.82 after tool use, indicating good internal consistency.

**Table S1**

*Embodiment Questionnaire in English*

| **Instructions** |
| --- |
| In reference to the preceding experimental block, please indicate whether there were moments for which you would agree with the following statements (from "*strongly disagree (1)*" to "*strongly agree (7)*"). |
| **Items** |
| - 1. I felt out of my body.   2. I felt as if my (real) body were drifting toward the virtual body or as if the virtual body were drifting toward my (real) body.   3. It felt as if my (real) body were turning into an “avatar” body.   4. At some point it felt as if my (real) body was starting to take on the posture or shape of the virtual body that I saw. *   5. I felt like I was wearing different clothes from when I came to the laboratory.   6. I felt as if my body had changed.   7. I felt that my own body could be affected by the virtual world.   8. I felt as if the virtual body was my body. *   9. At some point it felt that the virtual body resembled my own (real) body, in terms of shape, skin tone or other visual features. *   10. I felt as if my body was located where I saw the virtual body. *   11. I felt like I could control the virtual body as if it was my own body. *   12. It seemed as if I felt the touch of the floor in the location where I saw the virtual feet touched. *   13. It seemed as if the touch I felt on my feet was caused by the virtual floor touching the virtual feet.   14. It seemed as if my feet were touching the virtual floor.   15. I felt as if I was in the virtual room.   16. I felt nauseous. |
| **Likert Scale** |

| strongly disagree | disagree | somewhat disagree | neither agree nor disagree | somewhat agree | agree | strongly  agree |
| --- | --- | --- | --- | --- | --- | --- |
| (1) | (2) | (3) | (4) | (5) | (6) | (7) |

*Note.* The original 16-item questionnaire was adapted by removing two questions (R3 and R8 from Peck and Gonzalez-Franco (2021)^5^) due to limited relevance and reported comprehension difficulties in a pilot respectively. Two new items were added to assess presence (Q15) and nausea (Q16). Participants responded on a 7-point Likert scale from “1 = strongly disagree” to “7 = strongly agree”. Ownership subscale items are marked with an asterisk.

**Table S2**

*Embodiment Questionnaire Translated into German*

| **Instruktionen** |
| --- |
| Geben Sie bitte in Bezug auf den vorhergehenden experimentellen Block an, ob es Momente gab, für die Sie den folgenden Aussagen zustimmen würden (von "*stimme überhaupt nicht zu (1)*" zu "*stimme vollkommen zu (7)*"). |
| **Fragen** |
| - 1. Ich hatte das Gefühl, nicht mehr in meinem Körper zu sein.   2. Ich hatte das Gefühl, als würde mein (realer) Körper auf den virtuellen Körper zudriften oder als würde der virtuelle Körper auf meinen (realen) Körper zudriften.   3. Ich hatte das Gefühl, als würde sich mein (realer) Körper in einen „Avatar“-Körper verwandeln.   4. Irgendwann hatte ich das Gefühl, dass mein (realer) Körper anfing, die Haltung oder Form des virtuellen Körpers anzunehmen. *   5. Ich hatte das Gefühl, dass ich andere Kleidung trug als zu dem Zeitpunkt, als ich das Labor betrat.   6. Ich hatte das Gefühl, dass sich mein Körper verändert hatte.   7. Ich hatte das Gefühl, dass mein eigener Körper von der virtuellen Welt beeinflusst werden konnte.   8. Ich hatte das Gefühl, dass der virtuelle Körper mein Körper ist. *   9. Irgendwann hatte ich das Gefühl, dass der virtuelle Körper meinem eigenen (realen) Körper ähnelt, in Bezug auf Form, Hautfarbe oder andere visuelle Merkmale. *   10. Ich hatte das Gefühl, dass sich mein Körper dort befand, wo ich den virtuellen Körper sah. *   11. Ich hatte das Gefühl, dass ich den virtuellen Körper kontrollieren konnte, als wäre es mein eigener Körper. *   12. Es schien, als ob ich die Berührung des Bodens an der Stelle spürte, an der ich die virtuellen Füße berührt sah. *   13. Es schien, als ob die Berührung, die ich an den Füßen spürte, durch die Berührung des virtuellen Bodens mit den virtuellen Füßen verursacht wurde.   14. Es schien, als ob meine Füße den virtuellen Boden berührten.   15. Ich hatte das Gefühl, mich in dem virtuellen Raum zu befinden.   16. Mir war übel. |
| **Likert Skala** |

| stimme überhaupt nicht zu | stimme nicht zu | stimme eher nicht zu | weder noch | stimme eher zu | stimme zu | stimme vollkommen zu |
| --- | --- | --- | --- | --- | --- | --- |
| (1) | (2) | (3) | (4) | (5) | (6) | (7) |

*Note.* The original 16-item questionnaire was adapted by removing two questions (R3 and R8 from Peck and Gonzalez-Franco (2021)^5^) due to limited relevance and reported comprehension difficulties in a pilot respectively. Two new items were added to assess presence (Q15) and nausea (Q16). Participants responded on a 7-point Likert scale from “1 = stimme überhaupt nicht zu” to “7 = stimme vollkommen zu”. Ownership subscale items are marked with an asterisk.

**Table S3**

*Gaming and VR Experience Questionnaire in German*

| **Fragen** |
| --- |
| - 1. Während der letzten 12 Monate, wie viel Zeit haben Sie im Durchschnitt pro Woche damit verbracht Videospiele zu spielen?  \| niemals \| weniger als 1 Stunde \| zwischen 1 und 3 Stunden \| zwischen 3 und 5 Stunden \| zwischen 5 und 10 Stunden \| mehr als 10 Stunden \| \| --- \| --- \| --- \| --- \| --- \| --- \| \|  \|  \|  \|  \|  \|  \|  - 1. Welche Art von Videospiel haben Sie in den letzten 12 Monaten am meisten gespielt (mehrere Antworten möglich)? - Shooter (Call of Duty, Halo, Overwatch, Counterstrike, Apex Legends ...) - Rollenspiele (Elden Ring, The Witcher, Elder Scrolls, Starfield ...) - Sport (FIFA, Mario Kart, Forza ...) - Platformer (Super Mario, Ratchet & Clank, Ori and the Blind Forest ...) - Strategie/Puzzle (Starcraft, Civilization, League of Legends ...) - Musik (Guiter Hero, Beat Saber, Just Dance ...) - Andere (Handyspiele, Browserspiele ...)   1. Besitzen Sie eine eigene Virtual Reality (VR) Brille (z.B. die gleiche oder eine ähnliche virtuelle Brille, zu der, die wir im Labor haben)?  \| ja \| nein \| \| --- \| --- \| \|  \|  \|  - 1. Wie viel Zeit haben Sie insgesamt damit verbracht eine Virtual Reality (VR) Brille zu benutzen?  \| niemals \| weniger als 1 Stunde \| zwischen 1 und 3 Stunden \| zwischen 3 und 5 Stunden \| zwischen 5 und 10 Stunden \| zwischen 10 und 30 Stunden \| mehr als 30 Stunden \| \| --- \| --- \| --- \| --- \| --- \| --- \| --- \| \|  \|  \|  \|  \|  \|  \|  \| \|  \|  \|  \|  \|  \|  \|  \| |

*Note.* Q2 was only shown if any option other than “niemals” was chosen in Q1. The average time spent gaming covariate was based on responses to Q1, whereas the total time spent using VR headsets was based on responses to Q4.

**Table S4**

*Gaming and VR Experience Questionnaire Translated into English*

| **Items** |
| --- |
| - 1. Over the last 12 months, how much time on average did you spend per week playing video games?  \| never \| less than 1 hour \| between 1 and 3 hours \| between 3 and 5 hours \| between 5 and 10 hours \| more than 10 hours \| \| --- \| --- \| --- \| --- \| --- \| --- \| \|  \|  \|  \|  \|  \|  \|  - 1. What type of video game did you play the most in the last 12 months (multiple answers possible)? - Shooter (Call of Duty, Halo, Overwatch, Counterstrike, Apex Legends ...) - Role-Playing (Elden Ring, The Witcher, Elder Scrolls, Starfield ...) - Sport (FIFA, Mario Kart, Forza ...) - Platformer (Super Mario, Ratchet & Clank, Ori and the Blind Forest ...) - Strategy/Puzzle (Starcraft, Civilization, League of Legends ...) - Music (Guiter Hero, Beat Saber, Just Dance ...) - Other (Mobile Games, Browser Games ...)   1. Do you have your own virtual reality (VR) headset (e.g. the same or similar virtual headset to the one we have in the lab)?  \| yes \| no \| \| --- \| --- \| \|  \|  \|  - 1. How much time in total did you spend using virtual reality (VR) headsets?  \| never \| less than 1 hour \| between 1 and 3 hours \| between 3 and 5 hours \| between 5 and 10 hours \| between 10 and 30 hours \| more than 30 hours \| \| --- \| --- \| --- \| --- \| --- \| --- \| --- \| \|  \|  \|  \|  \|  \|  \|  \| \|  \|  \|  \|  \|  \|  \|  \| |

*Note.* Q2 was only shown if any option other than “never” was chosen in Q1. The average time spent gaming covariate was based on responses to Q1, whereas the total time spent using VR headsets was based on responses to Q4.

**Supplementary References**

1. Verrillo, R. T. Age related changes in the sensitivity to vibration1. *J. Gerontol.* **35**, 185–193, https://doi.org/10.1093/geronj/35.2.185 (1980).

2. Ekman, L., Lindholm, E., Brogren, E. & Dahlin, L. B. Normative values of the vibration perception thresholds at finger pulps and metatarsal heads in healthy adults. *PLOS ONE* **16**, e0249461, https://doi.org/10.1371/journal.pone.0249461 (2021).

3. Seidler, R. D., Alberts, J. L. & Stelmach, G. E. Changes in multi-joint performance with age. *Motor Control* **6**, 19–31, https://doi.org/10.1123/mcj.6.1.19 (2002).

4. Seidler, R. D. *et al.* Motor control and aging: links to age-related brain structural, functional, and biochemical effects. *Neurosci. Biobehav. Rev.* **34**, 721–733, https://doi.org/10.1016/j.neubiorev.2009.10.005 (2010).

5. Peck, T. C. & Gonzalez-Franco, M. Avatar embodiment. A standardized questionnaire. *Front. Virtual Real.* **1**, https://doi.org/10.3389/frvir.2020.575943 (2021).
